# Extra-articular Manifestations of Chikungunya

**DOI:** 10.1590/0037-8682-0341-2023

**Published:** 2023-12-08

**Authors:** Jobson Lopes de Oliveira, Igor Albuquerque Nogueira, J. Kennedy Amaral, Luciana Ribeiro Campos, Mariana Macêdo Militão Mendonça, Marília de Brito Ricarte, Luciano Pamplona de Góes Cavalcanti, Robert T. Schoen

**Affiliations:** 1 Universidade Federal do Ceará, Faculdade de Medicina, Departamento de Medicina Clínica, Fortaleza, CE, Brasil.; 2 Centro Universitário Christus, Faculdade de Medicina, Fortaleza, CE, Brasil.; 3 Instituto de Medicina Diagnóstica do Cariri, Juazeiro do Norte, CE, Brasil.; 4 Universidade de Fortaleza, Faculdade de Medicina, Fortaleza, CE, Brasil.; 5 Universidade Federal do Ceará, Faculdade de Medicina, Departamento de Saúde Comunitária, Fortaleza, CE, Brasil.; 6 Yale University School of Medicine, Section of Rheumatology, New Haven, CT, USA.

**Keywords:** Chikungunya, Arbovirus infection, Cardiovascular abnormalities, Kidney diseases, Neurologic manifestations, Skin manifestations

## Abstract

Chikungunya fever (CHIK) is a neglected tropical disease associated with chronic arthritis. CHIK is usually a self-limiting condition; however, extra-articular manifestations present as atypical illness in a minority of patients. These atypical features may mimic other conditions and potentially distract physicians from the true diagnosis. This review analyzes the evidence of many unusual extra-articular manifestations reported in cases of CHIK. Depending on the affected system, these unusual manifestations include encephalitis, myocarditis, acute interstitial nephritis, cutaneous manifestations, acute anterior uveitis, abdominal pain, and depression. In addition, coinfections and comorbidities may cause atypical illness and obscure the diagnosis. Further studies are required to clarify the pathophysiology and natural history of CHIK, as it remains a burdening condition. Exploring its atypical symptoms may be the missing scientific piece of this puzzle.

## INTRODUCTION

The chikungunya virus (CHIKV) is a mosquito-transmitted arthritogenic virus responsible for outbreaks of an acute febrile syndrome called chikungunya fever (CHIK). There are three distinct clades: the West African, Asian, and East/Central/South African (ECSA) genotypes. Most patients develop prominent symptoms of arthritis^1^. CHIK is usually self-limiting; however, some patients may present with atypical symptoms other than fever and arthralgia, potentially leading to fatal outcomes or leave long-term sequelae, such as encephalitis, myocarditis, nephritis, and deforming skin lesions^2^. The frequency of these unusual manifestations varies in the literature, ranging between 5% and 80%. Notably, older patients, pregnant women, and newborns exhibit higher susceptibility to these more severe presentations^3^ ^-^ ^6^.

When faced with atypical symptoms, diagnosing CHIK is more challenging. Given that CHIK is a severely neglected tropical disease, few studies have adequately characterized these atypical cases^1^. The quality and rarity of available data often limit existing reviews. This scarcity of data affects disease management, as physicians lack evidence to support specific treatment strategies and are compelled to treat patients empirically. Complications in the treatment of these unusual CHIK patients are also poorly described, and the impact of comorbidities on disease expression is also not well understood^7^. 

In this narrative review, we outline the current knowledge regarding the atypical, often severe, extra-articular presentations of CHIK infection, providing valuable insights into its pathophysiology, clinical picture, and management. Additionally, we discuss the prognoses of these manifestations and highlight the challenges and perspectives in managing possible sequelae. 

**FIGURE 1:**
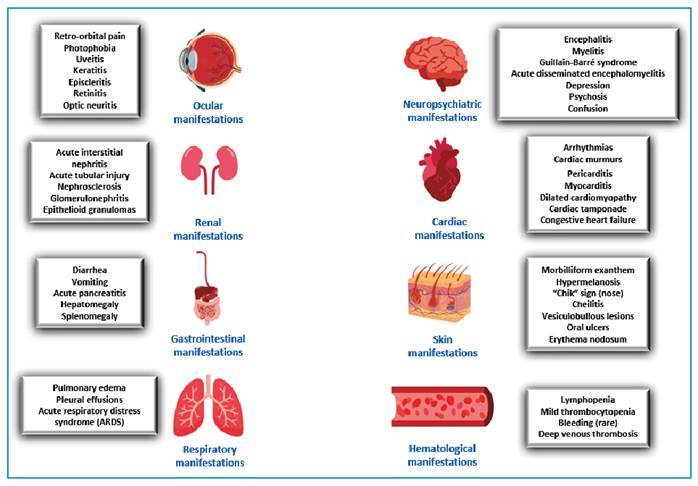
Overview of extra-articular manifestations of Chikungunya fever.

Figure 1 shows the main extra-articular manifestations of CHIK, which will be discussed below. 

## NEUROLOGICAL MANIFESTATIONS

Although CHIKV is not typically considered a neurotropic virus, sporadic cases of nervous system involvement have been reported since the 1960s^8^. The neurovirulence of the Asian genotype is believed to be the highest among the viral strains^9^.

The frequency of nervous system involvement in patients with CHIK ranges from 7 to 33 %. The most frequently observed complication is encephalitis, followed by myelitis, encephalomyelitis, Guillain-Barré syndrome, acute disseminated encephalomyelitis, optic neuropathy, and neuroretinitis. Less frequently reported complications include seizures, sensorineural hearing loss, stroke, cerebellitis, meningism, cranial nerve palsy, carpal tunnel syndrome, ophthalmoplegia, and disorientation^8^ ^,^ ^10^ ^-^ ^14^.

Previous studies have documented that children are more susceptible to neurological disease than adults^8^ ^,^ ^11^ ^,^ ^15^. Maternal-fetal transmission is a major risk factor for the development of neuro-Chikungunya, resulting in neurological symptoms and neurodevelopmental delay in up to 50% of infected neonates^16^.

Coinfection with other arboviruses, such as Zika and dengue viruses, is associated with more severe neurological disease, necessitating intensive care support and prolonged hospitalization. Additionally, cerebrovascular disease is three times more common in patients with dual infections than in those with mono-infection^11^. 

Treatment of CHIK neurological disease is typically symptomatic. The prognosis is variable, although most patients with neurological disease recover without sequelae. During CHIK infection, multiple organ systems can be simultaneously involved with neurological disease; therefore, a multidisciplinary approach is critical^9^.

## CARDIOVASCULAR MANIFESTATIONS

Cardiovascular manifestations are one of the most common extra-articular features of CHIK infection, affecting up to 54.2% of the infected patients^17^. The mechanism of cardiac involvement in CHIK remains unclear and may be multifactorial. In some patients, cardiac symptoms are primarily related to CHIK infection; however, in others, cardiovascular disease may be secondary to pre-existing comorbidities^3^. Animal models have revealed active replication of CHIKV in the hearts of immunodeficient mice, and human biopsies have shown CHIKV in the myocardium, suggesting that direct viral infection plays a pathogenic role in CHIK cardiac disease^18^ ^,^ ^19^. 

The protean cardiovascular manifestations of CHIK include pericarditis, cardiac tamponade, hypotension, shock, Raynaud’s phenomenon, arrhythmias, cardiac murmurs, myocarditis, dilated cardiomyopathy, congestive insufficiency, and heart failure. Approximately 22% of fatal CHIK cases are caused by cardiovascular diseases, with heart failure being the most common. The most commonly reported abnormality in the electrocardiograms of patients with CHIK is the inversion of T waves in leads DII, III, aVF, V5-V6, and elevation of the ST segment^20^ ^-^ ^22^. Most patients who do not die from acute cardiac involvement completely recover; however, case reports show that cardiovascular compromise can cause subacute and chronic illness, emphasizing the need for longitudinal recognition and management of patients with atypical CHIK^23^ ^-^ ^24^.

As with other arboviral infections, the optimal management of cardiovascular complications in patients with CHIK remains uncertain. The sporadic nature of cardiac CHIK cases limits these studies. Some unanswered questions include the lack of data on whether different CHIK viral strains affect cardiac susceptibility. From a treatment perspective, similar to other forms of viral myocarditis, the role of corticosteroids remains uncertain. The most crucial steps in management involve early monitoring and rapid intervention to prevent sequelae^19^ ^,^ ^20^ ^,^ ^25^ ^,^ ^26^. 

## HEMATOLOGIC MANIFESTATIONS

CHIKV infection causes milder laboratory abnormalities compared to infection. While mild thrombocytopenia may occur, severe cases are rare, with platelet levels usually above 100,000/µl. Lymphopenia is the most common abnormality, observed in approximately 80% of patients. Hemorrhagic complications are rare in CHIKV infection^27^ ^-^ ^29^. A relationship with deep venous thrombosis has also been proposed, with one study showing increased D-dimer levels in 63.8% of patients^30^. 

## RENAL MANIFESTATIONS

Renal complications have been reported in 21%-45% of CHIK cases and are more common among severely ill patients. Most of these patients have renal comorbidities, particularly chronic kidney disease, which contributes to high mortality rate^31^. It is still unknown whether the kidneys serve as reservoirs of the virus^32^. However, one study that evaluated patients with post-mortem biopsy-proven kidney injury established after CHIK infection did not detect viral antigens in the kidneys^33^. 

Acute interstitial nephritis and tubular injury were the most common renal findings^3^ ^,^ ^31^. Nephrosclerosis, membranoproliferative glomerulonephritis, and epithelioid granulomas have been less frequently reported. Among patients with acute interstitial nephritis, high serum creatinine levels manifest as impaired renal function^34^. In one study that evaluated co-infection with other arboviruses, renal manifestations were uncommon and did not affect prognosis^35^.

Kidney transplant recipients with CHIKV infection have a similar prognosis and clinical course as the general population. Although transitory graft dysfunction has been previously reported, it is usually mild and reversible^34^ ^,^ ^36^ ^,^ ^37^. 

The treatment of renal complications is symptomatic. In more severe cases, intensive care and monitoring of renal function may be imperative^38^. 

## CUTANEOUS MANIFESTATIONS

The most prevalent skin finding in CHIK infection is morbilliform exanthem, which is usually observed between the third and fifth days of febrile acute illness. It commonly starts in the upper limbs, spares the face, is self-limiting, and associated with mild pruritus. Immune complex deposition in the dermal capillaries is believed to be the pathophysiological mechanism underlying these cutaneous manifestations of CHIK. This type III hypersensitivity reaction may be observed in approximately 40-50% of cases^39^. 

Soon after the eruptions resolve, post-inflammatory hypermelanosis may occur, which is particularly common in dark-skinned individuals. Case reports have observed this skin condition in the nose, whereas some refer to this finding as a “CHIK sign.” The outer ear and limbs may also have been affected. Hypermelanosis can present as flagellated, freckle-simile, or discrete macules^40^ ^,^ ^41^.

**FIGURE 2:**
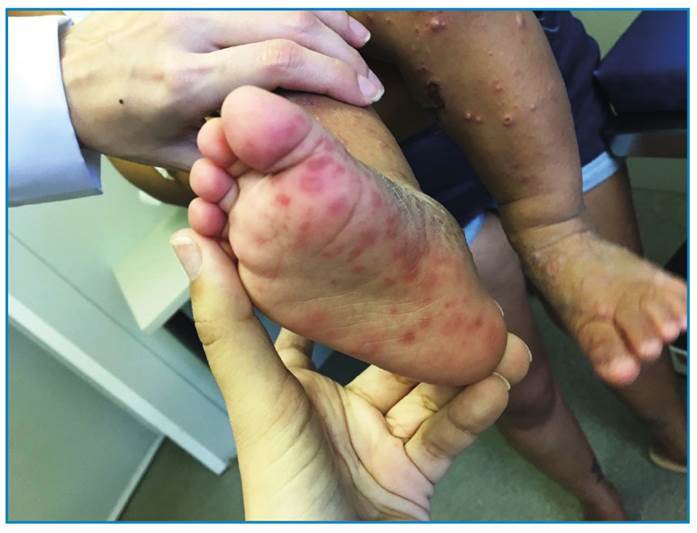
A 2-year-old patient with erythematous papules and plaques on the plantar region. (Photo courtesy of dermatologist Dr. Amanda Dantas).

**FIGURE 3:**
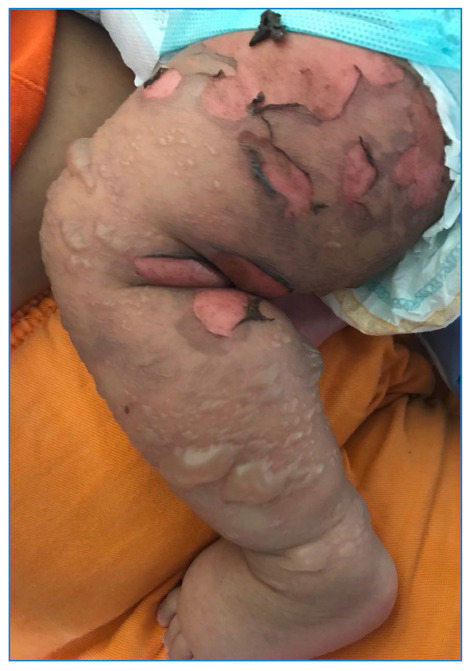
An 8-month-old patient with numerous flaccid blisters, some confluent, located on the lower limbs, with areas of exulceration with hematic crusts on the edges of the lesions. (Photo courtesy of dermatologist Dr. Amanda Dantas).

Exacerbation of preexisting dermatological conditions has also been reported in patients with CHIK. Erythroderma and psoriasis are the most common skin comorbidities that worsen during early febrile illness. Cheilitis, skin and mouth ulcers, crusted lesions, vesiculobullous lesions (frequently observed in children and neonates), skin peeling, xerosis, papules, erythema nodosum, and vasculitis-like lesions have also been reported^42^ ^-^ ^44^. CHIKV may also act as a trigger for vitiligo and other autoimmune diseases^45^ ^,^ ^46^. Figures 2 and 3 show examples of cutaneous manifestations of CHIK.

Nail disorders in CHIK occur as red or black lunulae, periungual ulcers, and/or subungual hemorrhage. Periungual desquamation, and diffuse and longitudinal melanonychia have also described^41^. 

Treatment of CHIK skin manifestations commonly relies on antibiotics (e.g., erythromycin) and corticosteroids. Although most skin lesions resolve spontaneously, some heal with significant scarring. Drug choices are inconsistent between studies, and there is limited evidence on the efficacy of these strategies, given the self-limiting character of skin findings^40^ ^-^ ^42^.

## OCULAR MANIFESTATIONS

Ocular manifestations of CHIK infection include nonspecific conjunctival conditions, retinitis, and occasional exudative retinal detachment^47^. The most common finding is acute nongranulomatous or granulomatous anterior uveitis; however, cases of intermediate, posterior, and panuveitis have also been observed. Episcleritis, optic neuritis, secondary glaucoma, keratitis, and nerve VI palsy are rarer^47^ ^,^ ^48^. It has not been established whether the ocular manifestations of CHIK are immune-mediated or directly caused by viral infection^49^ ^,^ ^50^.

During the acute phase of the disease, retro-orbital pain and photophobia without visual changes are the most prevalent ocular symptoms. Throughout the illness, patients may report decreased visual acuity, which is a symptom that causes most patients to seek ophthalmological evaluation. Unilateral or bilateral uveitis, ocular hypertension, retinal edema, and retinal hemorrhage may cause decreased visual acuity, and more rarely, inflammatory foveal lesions and macular ischemia^47^ ^,^ ^50^ ^,^ ^51^.

The treatment for uveitis generally involves the use of corticosteroids. A favorable response may require 10-12 weeks. The best response is obtained when the diagnosis is promptly made and treatment is immediately initiated^52^.

## GASTROINTESTINAL MANIFESTATIONS

Gastrointestinal symptoms, especially abdominal pain and nausea, have been reported in up to 66% of patients^53^. However, the available data vary depending on the study population. Diarrhea, vomiting, acute pancreatitis, hepatomegaly, and splenomegaly have been observed in a minority of patients in some reports^54^ ^,^ ^55^. Patients with the Asian strain of CHIK seem to present more visceral enlargement than other patients; however, the prevalence of other gastrointestinal symptoms is similar^55^. Hepatitis, which manifests as abnormal asymptomatic levels of liver enzymes, is usually mild^56^. In one study, women experienced more nausea and abdominal pain compared to men^54^.

Conclusive information on gastrointestinal symptoms in CHIK remains unattainable because of the low number of publications on the subject and differences in the populations studied and the prevalent strains. There is no consensus regarding the treatment of the gastrointestinal manifestations of CHIK. 

## PSYCHIATRIC MANIFESTATIONS

In some patients with neurological involvement, there may be psychiatric symptoms such as confusion, delirium, disorientation, and even psychosis^57^. However, few studies have reported specific psychiatric manifestations of acute CHIK, including mania and depression^58^ ^-^ ^60^. Mania has been reported in a patient with acute CHIK who was previously diagnosed with bipolar disorder, although the patient was stabilized with valproic acid^58^. Another study reported a CHIK-induced manic episode in a patient with no psychiatric history^59^.

Depression in acute CHIK affects up to half of the patients. Younger patients and those with severe arthritis and gastrointestinal symptoms are more likely to self-report depression^60^ ^.^ ^61^. 

In acute CHIKV infections, elevated levels of cytokines, including IL-6, are associated with stress susceptibility along with social factors that contribute to post-CHIKV depression. Notably, IL-6, IL-1RA, IL-12, TNF-α, IFN-γ, IL-10, IL-1β, and IL-8/CXCL8 exhibit increased levels in both acute and chronic CHIKV cases, as well as in depression. This suggests the potential of concurrent immunomodulatory interventions targeting these cytokines in comprehensive clinical assessments to mitigate the impact of CHIKV and depressive disorders^62^ ^.^ ^63^.

Manic episodes are treated with antipsychotics such as olanzapine^58^ ^.^ ^59^. The treatment of depression is similar to that used in other settings^60^ ^.^ ^63^. As psychiatric manifestations correlate with pain, analgesics may help treat chronic CHIK infections. Multifactorial and holistic approaches are major elements of optimal care, given that few pharmacological therapies effectively reduce the burden of chronic CHIK^63^.

## RESPIRATORY MANIFESTATIONS

Although rare, respiratory manifestations can occur in CHIK patients. CHIKV can infect the lungs and the CHIKV antigen has been isolated from post-mortem biopsies. An intense type I inflammatory response to the virus can lead to acute respiratory distress syndrome (ARDS)^64^. Pulmonary edema and pleural effusion may occur because of endothelial cell dysfunction^65^. Respiratory complications in CHIK patients are strongly associated with higher mortality rates. Pneumonia and respiratory failure are reported as common causes of death in atypical severe CHIK^65^ ^,^ ^66^. Concomitant bacterial infections and pre-existing comorbidities such as emphysema and asthma are believed to increase mortality^66^.

Management of respiratory manifestations is mostly supportive. Noninvasive ventilation may be used successfully in ARDS^67^. 

## CONCLUSION

Although CHIK infection is usually self-limiting, extra-articular involvement with atypical symptoms may present diagnostic challenges, delay diagnosis, and negatively affect disease outcomes. According to the affected system, the main extra-articular manifestations include encephalitis, myocarditis, acute interstitial nephritis, maculopapular rash, acute anterior uveitis, abdominal pain, and depression. Co-infections, comorbidities, advanced age, pregnancy, and newborns are significant risk factors for unusual and more severe manifestations. 

The pathogenesis, clinical features, and management of extra-articular CHIK infections remain unclear. Understanding the pathogenic mechanisms underlying CHIK could also be applicable to other rheumatological and autoimmune diseases, given the role of inflammation and cytokines. An improved understanding of the clinical features of extra-articular manifestations could lead to timely recognition of CHIK, reducing the incidence of its potential complications and long-term sequelae, which represent a high burden to patients. Potential treatments for this neglected tropical disease should be developed and made available in the endemic regions. Such treatments must consider the socioeconomic factors that impact the disease toll. Public health policies in settings where CHIK is prevalent must consider the priorities and values of individuals and their cultural beliefs in order to reduce inequalities, achieve better access to care, and improve the overall quality of life. 
